# Sleep onset is a creative sweet spot

**DOI:** 10.1126/sciadv.abj5866

**Published:** 2021-12-08

**Authors:** Célia Lacaux, Thomas Andrillon, Céleste Bastoul, Yannis Idir, Alexandrine Fonteix-Galet, Isabelle Arnulf, Delphine Oudiette

**Affiliations:** 1 Sorbonne Université, Institut du Cerveau - Paris Brain Institute - ICM, Inserm, CNRS, Paris 75013, France.; 2 Monash Centre for Consciousness and Contemplative Studies, Faculty of Arts, Menzies Building, 20 Chancellors Walk, Clayton Campus, Monash University, Melbourne, VIC 3800, Australia.; 3 AP-HP, Hôpital Pitié-Salpêtrière, Service des Pathologies du Sommeil, National Reference Centre for Narcolepsy, Paris 75013, France.

## Abstract

The ability to think creatively is paramount to facing new challenges, but how creativity arises remains mysterious. Here, we show that the brain activity common to the twilight zone between sleep and wakefulness (nonrapid eye movement sleep stage 1 or N1) ignites creative sparks. Participants (*N* = 103) were exposed to mathematical problems without knowing that a hidden rule allowed solving them almost instantly. We found that spending at least 15 s in N1 during a resting period tripled the chance to discover the hidden rule (83% versus 30% when participants remained awake), and this effect vanished if subjects reached deeper sleep. Our findings suggest that there is a creative sweet spot within the sleep-onset period, and hitting it requires individuals balancing falling asleep easily against falling asleep too deeply.

## Teaser

A single minute of sleep onset is sufficient to boost the probability of insight by a factor of three compared to wake.

## INTRODUCTION

A few empirical studies have shown that sleep helps extract statistical regularities (*1*–*3*), solve problems (*4*, *5*), or reorganize associative memories in a way that promotes creativity (*6*–*8*). Although the role of sleep in creative problem-solving is oft cited in the sleep literature (*9*, *10*), supporting evidence is unexpectedly scarce when compared to other cognitive functions like memory consolidation (*11*). Most existing research has focused on the role of rapid eye movement (REM) sleep and slow-wave sleep in creative problem-solving, which would respectively foster distant semantic associations and gist abstraction (*10*). Contrary to other sleep stages, the first stage of non-REM sleep (N1) has received little attention, and its cognitive role is largely unknown. Yet, a recent study showed that 10 min of “awake quiescence” (i.e., a quiet rest spent in a dimly lit room with reduced sensory stimulation) more than doubled the number of subjects who discovered a hidden rule compared to 10 min of active wake (*12*). Such conditions are likely to foster brief intrusions into N1 (*13*, *14*), but this possibility was not investigated.

Nonetheless, we believe that N1 presents an ideal cocktail for creativity. Creative cognition is supposed to rely on a dynamic interplay between brain networks involved in spontaneous thinking (default mode network) and cognitive control, which respectively support creative idea generation and evaluation (*15*). Neuroimaging studies of the sleep onset period have shown that N1 precisely engages these networks instrumental to creativity (*16*, *17*). In addition, N1 is accompanied by involuntary, spontaneous, dream-like perceptual experiences (*18*, *19*) that incorporate recent wake experiences (*20*, *21*) in a creative way by binding them with loosely associated memories (*22*). Such hypnagogic experiences could be considered as an exacerbated version of awake spontaneous thoughts (e.g., mind-wandering) (*23*, *24*) and similarly foster the generation of novel ideas (*25*). In line with this hypothesis, we recently reported an increased creative potential in patients with narcolepsy (*26*), a population with frequent transitions toward sleep during the day. A subsequent study further identified hypnagogic hallucinations as a key modulator of creativity in narcolepsy (*27*). On the other hand, thalamic deactivation in N1 often precedes that of the cortex by several minutes (*28*), suggesting that executive abilities are not completely abolished during this stage. Consistently, subjects are sometimes capable of producing behavioral responses in N1 (*29*) and often unexpectedly report that they were awake when awakened from N1 (*30*, *31*). These observations support the view that N1 is a hybrid, “semilucid” state where individuals start to be decoupled from their environment and can therefore freely watch their minds wander while maintaining their logical ability to identify creative sparks (*32*).

**Fig. 1.**
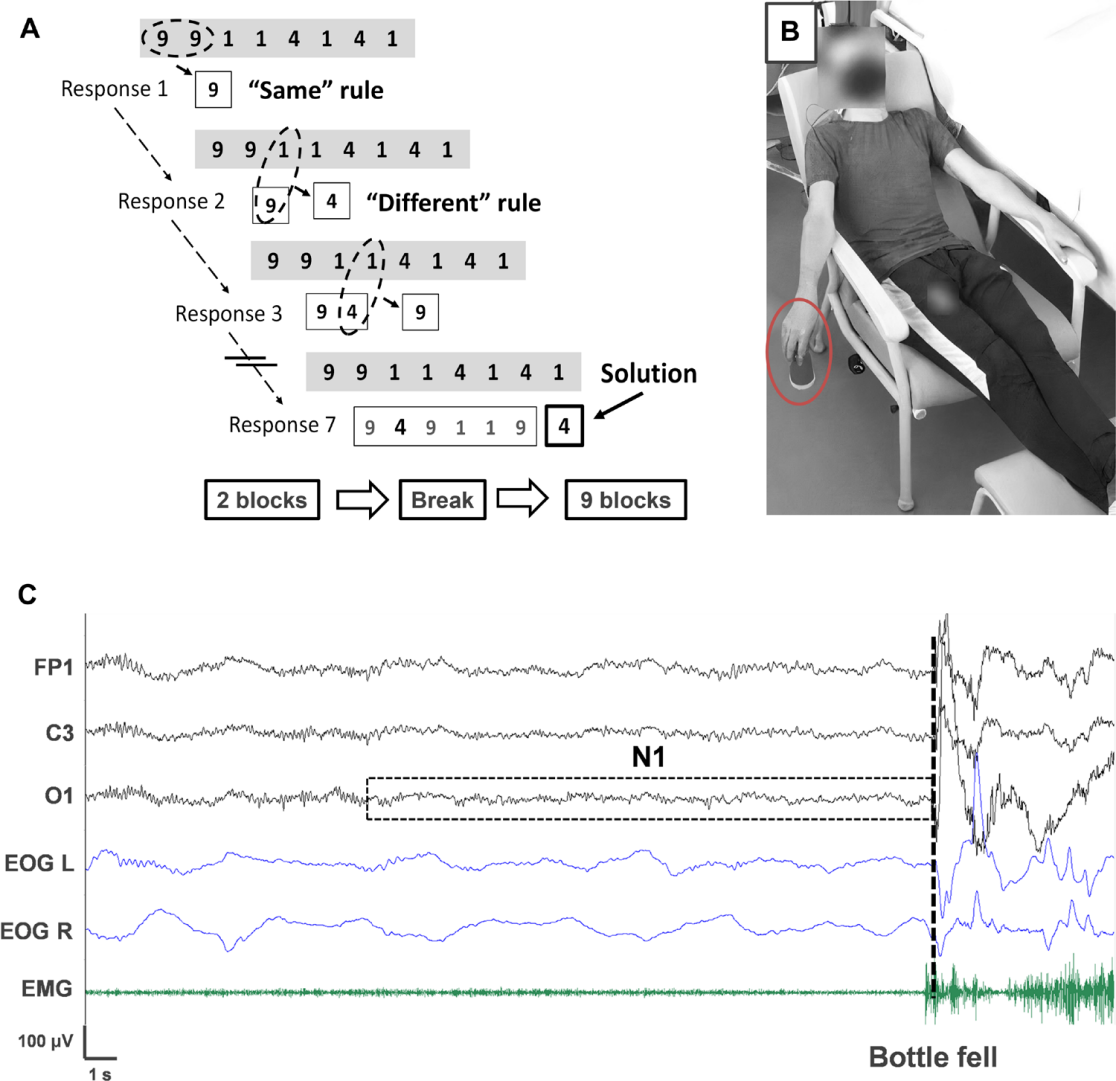
Experimental paradigm. (**A**) Top: An illustrative NRT trial. Subjects were presented with an eight-digit sequence (composed of 1, 4, and 9) and instructed to find the final solution as quickly as possible by applying two rules in a stepwise manner: Report the same digit if the previous and next digits are identical (same rule) and the remaining third digit if they are different (different rule). Unbeknownst to them, the second response was always the final solution (hidden rule allowing them to answer much faster). Adapted from (*1*). Bottom: Protocol timeline. Subjects completed two blocks (30 trials each) of the NRT and then had a 20-min break followed by nine additional blocks. (**B**) Break period. Participants rested in a chair, eyes closed, in a dark room while holding a bottle (circled in red) in their right hand. They were told to report out loud any mental content if the object fell. (**C**) Illustrative polysomnographic recording. In this example, the participant was in N1 when the bottle fell (dashed line), waking him up. He then reported a hypnagogic experience [“I saw a big cliff and I was climbing it. Then boom, it (the bottle) fell and brought me back to reality”].

Here, we tested whether a brief period of N1 fosters creative insight, defined herein as the sudden discovery of a solution to a problem (*1*). We used the number reduction task (NRT) (*33*), which has been shown to reliably capture an incubation-related gain of insight following a full night of sleep (*1*) or 10 min of wake quiescence (*12*). At each trial, subjects (*N* = 103) were given strings of eight digits and instructed to reach a final digit solution as quickly as possible. To do so, they were informed that applying two simple rules in a stepwise manner would lead to the solution. Unbeknownst to them, a hidden rule permitted them to shortcut the series of operations and obtain the solution much faster (see Fig. 1A for more details). After a short training, all participants completed two blocks of 30 trials each (Pre phase, Fig. 1A). Participants who gained insight into the hidden rule at that early phase (15.53%, 16 of 103) were excluded from subsequent analyses. Then, participants had a 20-min break (Break phase) during which they were asked to relax in a semi-reclined position with their eyes closed. Participants were instructed to hold an object in their right hand and, if the object were to fall, to report out loud their stream of thoughts just before the fall (see Fig. 1, B and C for an illustration). Such a procedure was inspired by the famous inventor Thomas Edison, who allegedly napped while holding spheres in his hands. He reckoned that the spheres would noisily drop as soon as he fell asleep, waking him up just in time to capture sleep-inspired ideas. Here, we tested Edison’s intuition that there is a fleeting, propitious moment for insightful thoughts within the sleep onset period (e.g., reaching the hypnagogic period, but awakening before deeper sleep arises). Throughout the break, participants’ vigilance state was monitored via video polysomnography [simultaneous electroencephalographic (EEG), electro-oculographic (EOG), and electromyographic (EMG) recordings]. After the break, subjects were tested again on nine blocks of NRT (Post phase).

## RESULTS

We scored each participant’s break using standard sleep scoring criteria (*34*) and divided participants into three groups based on their vigilance state during the break (see demographic and sleep parameters in table S1): the “Wake” group (subjects who stayed awake for the entire break duration, *N* = 49), the “N1” group (subjects with at least one 30-s epoch of N1 but without any signs of deeper sleep stages, *N* = 24), and the “N2” group (subjects who had at least one 30-s epoch of NREM sleep stage 2 or N2, *N* = 14, including three who directly fell into N2 without passing by N1). Critically, all groups were exactly in the same conditions during the incubation period. Subjects in the N1 group were awake most of the time, spending only 1 min in N1 (1.35 ± 1.20 min); subjects in the N2 group spent a similar time in N1 than the N1 group (mean = 1.79 min ±2.11, Mann-Whitney, *U* = 156.5, *z* = −0.33, *P* = 0.73) plus an average of 4.18 (±2.94) min in N2.

### A single minute of N1 inspires insight

**Fig. 2.**
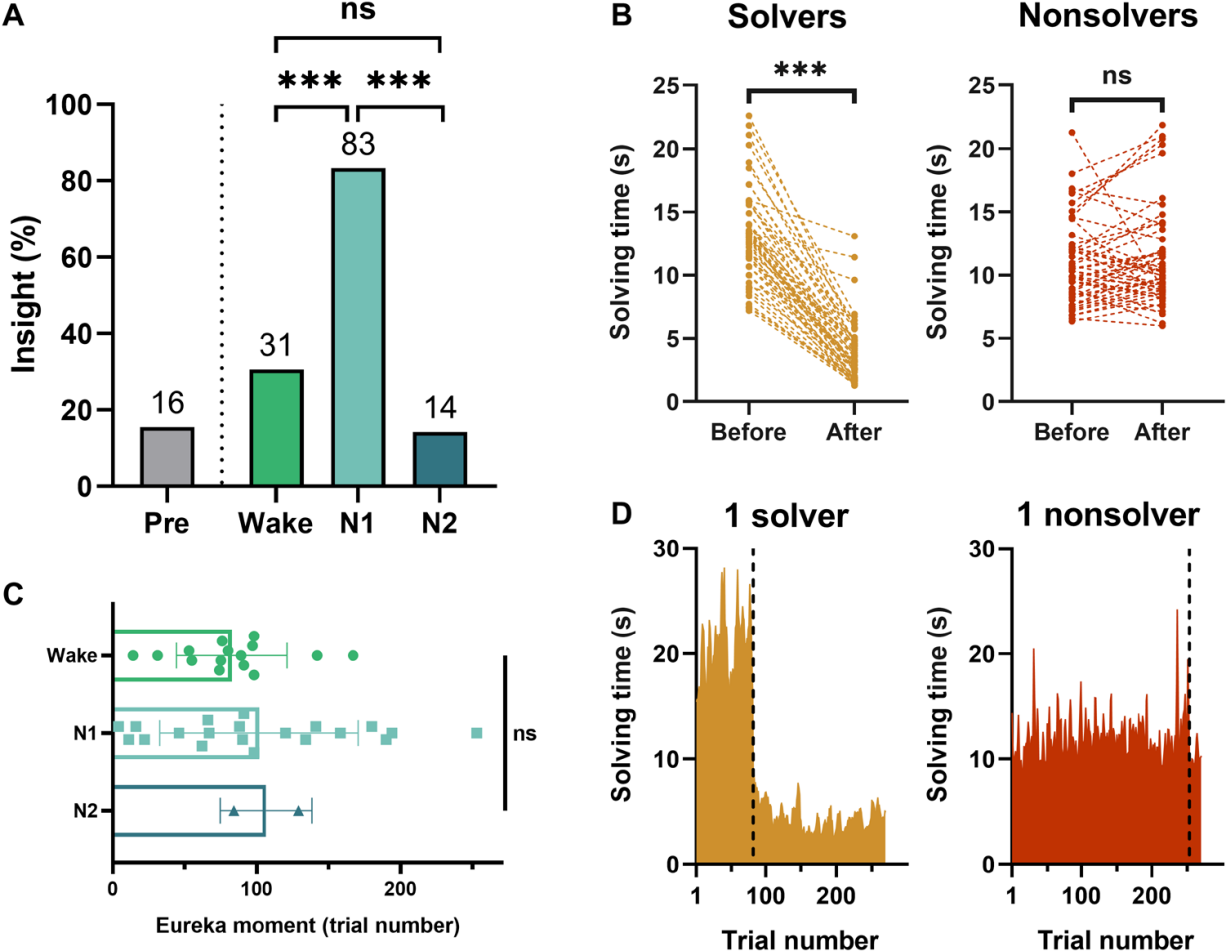
N1 inspires insight. (**A**) Percentage of solvers before sleep (all groups) and after sleep (Wake, N1, and N2 groups defined according to their polysomnographic activity during the break, *N*_Pre_ = 103, *N*_wake_ = 49, *N*_N1_ = 24, *N*_N2_ = 14). (**B**) Solving time before and after the Eureka moment for all solvers (*N* = 53) and nonsolvers (*N* = 50). Each dashed line represents a subject. Note that four solvers did not exhibit a clear shift in performance before and after the identified Eureka moment. They explicitly reported the hidden rule during debriefing and explained that they did not use it as they considered it as cheating. (**C**) Trial number at which the Eureka moment occurs during the Post phase. (**D**) Illustration of the Eureka moment (dark dashed line) automatically detected by an algorithm that identifies where the most abrupt change in solving time lies for both a solver (left) and a nonsolver (right). ***P < 0.001; n.s. for nonsignificant differences (Fisher test for comparisons of proportions between groups, Wilcoxon signed-rank test for comparisons between two paired samples: solvers and nonsolvers, and Kruskal-Wallis test for comparisons between all three groups).

We found a significant effect of the group (wake versus N1 versus N2) on the percentage of participants who found the hidden rule after the break (Fisher’s test, *P* < 0.001; Fig. 2A). This effect was driven by the percentage of insight in the N1 group, which was 2.7 times higher (83.33%, 20 of 24 subjects) than in the Wake group (30.61%, 15 of 49; compared with N1 versus Wake: Fisher’s test, *P* < 0.001) and 5.8 times higher than in the N2 group (14.29%, 2 of 14, compared with N1 versus N2: Fisher’s test, *P* < 0.001). The percentage of individuals gaining insight was similar in the Wake and N2 groups (Fisher’s test, *P* = 0.32). Of note, only 3 of the 16 participants who found the rule before the break (excluded from the previous and remaining analyses) slept in N1 (without any signs of N2), strongly suggesting that there is no a priori relationship between a specific sleep/wake trajectory during the nap and general insight abilities.

### A delayed Eureka moment

Subjects explicitly reported insight into the hidden rule at the end of the experiment, and insight was confirmed by a steep decrease in solving time after the Eureka moment (mean Before = 13.07 ± 3.71 s, After = 3.81 ± 2.46, Wilcoxon signed-rank test, *z* = −6.33, *P* < 0.001; Fig. 2B) and accompanied by an increase of accuracy (mean Before = 91.15 ± 6.73%, After = 96.86 ± 3.71%, Wilcoxon signed-rank test, *z* = 5.05, *P* < 0.001) for solvers only (see fig. S1 for details on accuracy and speed-accuracy trade-off). The “Eureka” moment was calculated by an algorithm (see Materials and Methods) that automatically detected abrupt changes in solving times across all trials from the Post phase. The same algorithm was used in nonsolvers for comparison with solvers and to confirm the absence of insight (see Fig. 2D for an illustrative example in one solver and one nonsolver). This Eureka moment did not occur immediately following the resting period, but rather after 94 trials on average, regardless of the group [mean Wake= 82.67, N1 = 101.55, N2 = 106.50, Kruskal-Wallis test: χ^2^(2) = 0.64, *P* = 0.73; Fig. 2C], a result consistent with previous studies using the NRT (*1*, *12*). Of note, we did not find evidence of an implicit knowledge of the rule in solvers before Eureka (see fig. S2).

Altogether, these results demonstrate that an incubation period containing a brief period of N1 has a marked effect on insight, but that this beneficial effect vanishes if participants reach a deeper state of sleep (N2). Control analyses suggest that the higher proportion of insight observed in the N1 group is not related to confounding factors, as all groups were indistinguishable in terms of initial performance, subjective vigilance, or motivation level (see table S1). Participants in the N2 group were slightly slower in the post-break psychomotor vigilance test (PVT) than the other groups (table S1). However, performance on the first NRT block of the Post phase was similar in the three groups (mean solving time: Wake = 10.6 s; N1 = 11.92 s; N2 = 11.29 s, Kruskal-Wallis test, *P* = 0.41), suggesting that any behavioral trace of sleep inertia quickly vanished after the PVT. This finding, along with the fact that the average length of the Post phase was approximately 1 hour (leaving plenty of time to discover the rule after the dissipation of sleep inertia), strongly suggests that the lower rate of insight in the N2 group cannot be explained by sleep inertia.

### Neurophysiology of the sweet spot

**Fig. 3.**
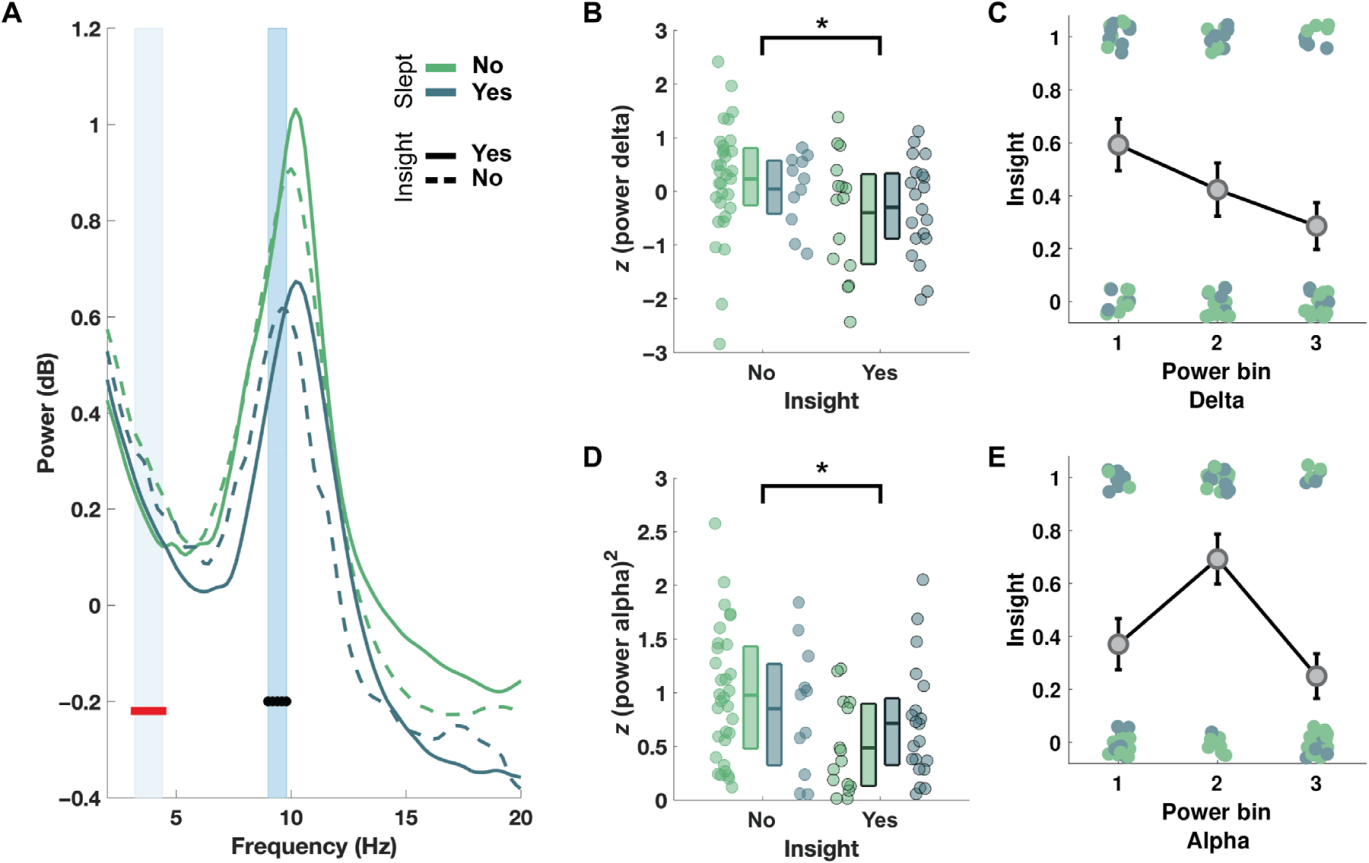
Alpha and delta power capture the sleep-onset creative sweet spot. (**A**) Average power spectrum over the occipital electrode during the break (*N* = 78 recordings) for participants who slept (blue) or not (green), further split between solvers (continuous line) and nonsolvers (dotted line). We identified two clusters of frequencies associated with insight, in the alpha ([9, 9.8] Hz, black line) and the delta bands ([3.2, 4.4] Hz, red line) (all *P*_cluster_ < 0.001). (**B** and **D**) Average power in the delta (*z*-scored across all participants) and alpha bands (squared *z*-score) for solvers (yes) or nonsolvers (no), further divided into participants who slept (blue) or not (green). *z*-score values show the linear effect of delta (ANOVA with between-subjects’ effects of sleep and insight; effect of insight: *P* = 0.019), whereas the squared *z*-score values show the quadratic effect of alpha (*P* = 0.013). Individual subjects are represented by circles. Horizontal lines show the first, mean, and third quartiles. *P < 0.05. (**C** and **E**) Same as (B) and (D), but delta and alpha power were binned into three levels, and the proportion of participants reaching insight (yes = 1 and no = 0) was computed for each bin (gray circles and error bars represent the mean and SEM).

Here, the twilight zone between wakefulness and sleep was lumped together as N1, following the standard approach in sleep research. However, sleep onset is a complex, dynamic process (*35*, *36*), potentially encompassing multiple transitions between different substages (*31*), each with subtle variations in physiological activity (e.g., alpha/theta, muscle relaxation). To better understand the critical factors for boosting insight, we calculated the power spectra for the entirety of the resting period and assessed whether it could predict subsequent Eureka moments in all subjects. These analyses revealed that the power in the delta (3.2 to 4.4 Hz) and alpha (9 to 9.8 Hz) bands were both predictive of insight (Fig. 3A). Precisely, we found a negative, quadratic effect of alpha power on insight (Fig. 3, A and D), meaning that subjects with intermediary levels of alpha power had the highest probability of insight (Fig. 3E). Furthermore, we observed a negative, linear effect of delta power on insight (Fig. 3, A and B), meaning that subjects with the highest levels of delta power had the lowest probability of insight (Fig. 3C). Crucially, the effect of delta and alpha power was assessed above and beyond each participant’s group (Wake, N1, or N2) and could therefore explain why some subjects who remained awake still discovered the hidden rule (i.e., wake solvers). Post hoc analyses in the Wake group show that wake solvers differed from their peers (i.e., wake nonsolvers) in that they had significantly lower levels of squared alpha power (unpaired Wilcoxon rank test, *W* = 355, *P* = 0.008) with a trend toward lower levels of delta power (*W* = 316, *P* = 0.08). Overall, these results point to the existence of a creative sweet spot within the sleep onset period, as well as the reasons why some participants failed to reach it. Reaching it requires a trade-off between the ability to fall asleep and attain N1 (which is favored by an intermediate level of alpha), but without too much sleep pressure to avoid transitioning into deeper sleep (associated with a low level of alpha and a high level of delta power).

To better understand the critical factors fostering insight, we fitted a series of logistic regression models with different variables of interest (see Materials and Methods). Using model comparison, we found that insight was best predicted [smallest Akaike information criterion(AIC)] by a model including fixed factors of sleep group, a linear effect of delta power, and a quadratic effect of alpha power (see Materials and Methods and table S2). Post hoc analyses indicate three significant effects: an effect of the sleep group [χ^2^(1)= 6.24, *P* = 0.013], a linear effect of delta power [χ^2^(1) = 5.99, *P* = 0.014], and a quadratic effect of alpha power [χ^2^(1) = 8.11, *P* = 0.004]. Thus, delta and alpha power can improve the characterization of the creative sweet spot beyond standard sleep stages.

### A reliable marker of sleep onset

**Fig. 4.**
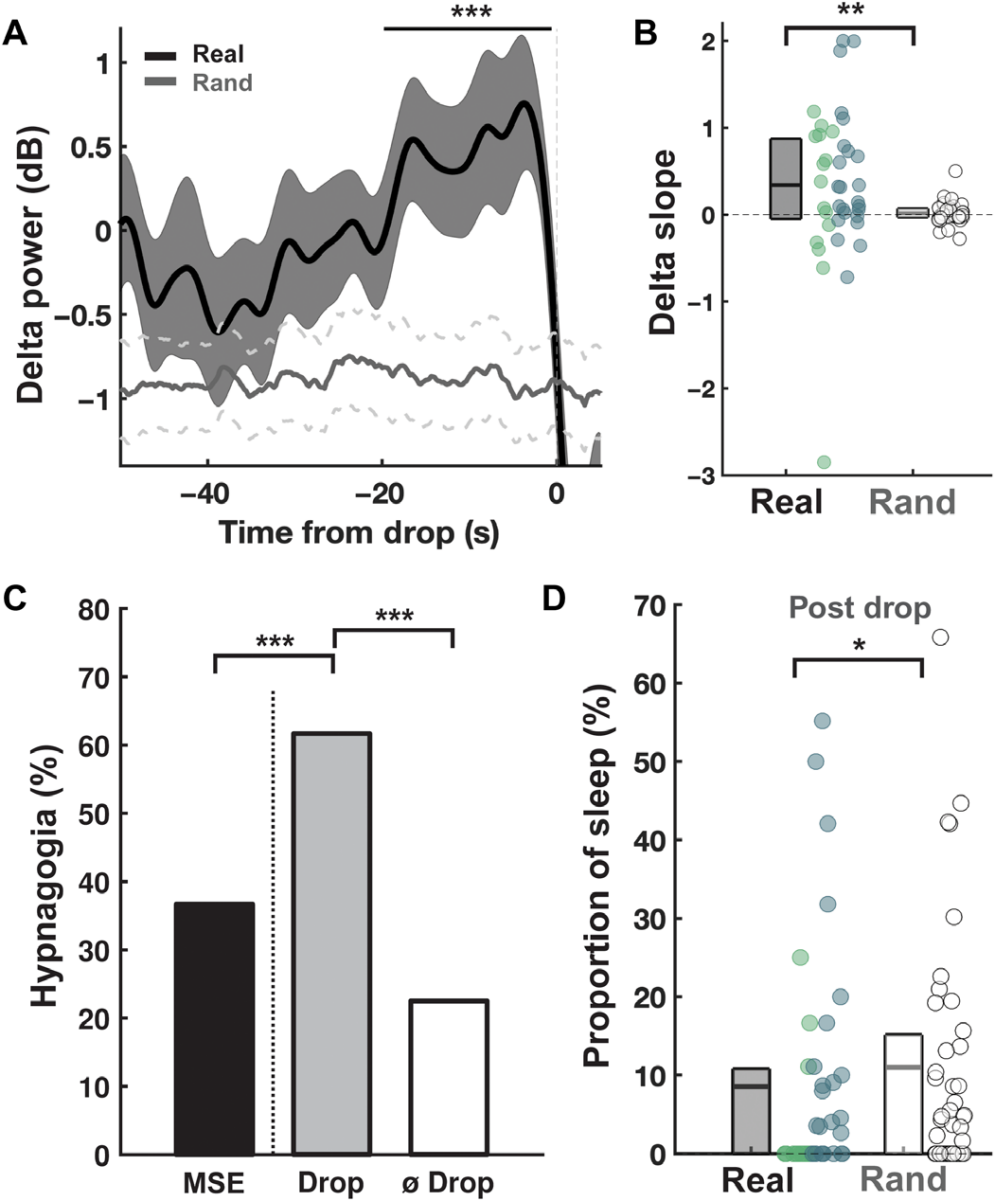
Edison’s technique is a reliable marker of sleep onset. (**A**) Average delta power around bottle drops ([−50, 10] s) in subjects who dropped the bottle (real, *N* = 39 individual recordings; when applicable, averaged across multiple drops, *N* = 8 of 39). To approximate what would have happened if bottle drops were independent of participants’ vigilance state, we show the corresponding average delta power when selecting 100 randomly drawn times for each recording (Rand). Shaded areas/dotted lines show the SEM and solid lines show the mean. The horizontal black line shows the significant cluster between real and rand data (*P* < 0.001). (**B**) A first-degree polynomial was fitted to the average delta power time course [−20, −2] s obtained for each recording and the corresponding slope is shown for real and random times. (**C**) Percentage of hypnagogic experiences collected after awakenings from MSE (control experiment) and in subjects with and without drop. ***P < 0.001 (chi-square test). (**D**) Proportion of 30-s sleep epochs (standard sleep criteria) after each drop for real and random times. (B and D) For real, circles denote subjects who slept/had MSE(s) (blue) or remained awake (without any microsleep, green). Horizontal lines show the first quartile, mean, and third quartiles. **P < 0.01; *P < 0.05 (Wilcoxon signed-rank test).

Last, we aimed to experimentally verify Edison’s intuition that holding an object while napping is propitious to capturing creative sparks (i.e., the object would drop and awaken the subject at the precise moment of the creative sweet spot). We first tested whether participants dropped the object when asleep. This does not appear to be entirely the case: Of the 63 drops, 26 (41.27%) occurred after N1. However, when we examine the wake-to-sleep transition taking into account microsleep episodes (MSEs), which are not considered in the standard sleep scoring method over 30-s-long windows (see Materials and Methods), this proportion rises to 77.78% (*N* = 49 of 63), with the bottle dropping after approximately 1 min of accumulated MSEs (table S1). The bottle drop thus appears more as a marker of the accumulation of MSEs rather than of consolidated N1. We further tested the ability of Edison’s method to capture sleep onset by performing spectral analyses restricted to the period around the bottle drop ([−50, −10] s). We observed a clear power increase in the delta band 24 s before the drops (significant cluster: [−23.8, −0.2] s, *P*_cluster_ < 0.0001; Fig. 4A), an increase that was superior to the one that would be observed by chance (randomly generated time drops, see Materials and Methods). This result was consistent regardless of the group, even in subjects who dropped the bottle in the absence of any MSEs (Fig. 4B). Of note, this delta increase was congruent with subjective reports as most subjects (81.48%) reported that they had been drifting to sleep when the bottle dropped. Edison’s technique also acted as an efficient “hypnagogia catcher” (see some examples in the Supplementary Materials) because the amount of collected hypnagogic experiences when the object dropped (61.70%, with 34.48% of those being task-related) was significantly higher than when it did not drop (22.50% collected at the end of the nap, χ^2^ = 13.50, *P* < 0.001), as well as higher than the percentage of hypnagogic experiences collected after awakenings from MSE in an additional control experiment (see the Materials and Methods for more information on that control experiment; mean cumulated time of MSE before awakenings = 1.10 min ± 1.08; 50 of 137 = 36.49% of awakenings with reported hypnagogia; χ^2^ = 12.80, *P* < 0.001; Fig. 4C). However, neither hypnagogic experiences in general (χ^2^ = 0.14, *P* = 0.71) nor task-related hypnagogia (χ^2^ = 0.05, *P* = 0.83) had an effect on insight.

Altogether, electrophysiological, behavioral, and subjective measures all point to the conclusion that Edison’s technique permits detecting fine-grained shifts toward sleep even in subjects who would normally be considered awake. Is this technique useful for fostering insight in addition to detecting sleep onset? We believe that it does to some extent: It does not aid in precisely capturing the creative sweet spot, but it does help in keeping subjects who have reached it in that zone. We demonstrated that the bottle is sensitive to the accumulation of delta power (Fig. 4, A and B), the same frequency that we found is detrimental for insight (Fig. 3). This observation, combined with the fact that the bottle always woke the subjects up (100%), suggests that it interrupted the buildup of delta and prevented subjects from reaching deeper sleep, which was deleterious for insight. Consistently, only 5 subjects of the 33 (15%) who fell asleep and dropped the bottle showed evidence of N2 before the first bottle drop. Furthermore, permutation analyses show that the proportion of sleep after after the bottle drop is lower than would be expected by chance if the bottle fell at random times (Fig. 4D).

## DISCUSSION

Together, our results demonstrate that the discovery of a hidden rule is 2.7 times more likely after spending only 1 min of N1 during an incubation period, compared to a similar period of quiet rest including only wakefulness. This facilitating role of N1 in insight disappeared when participants reached deeper sleep. Spectral analyses substantiate these findings and unravel a “creative sweet spot,” consisting in a medium level of alpha and a low level of delta. This creative sweet spot largely overlaps with the standard N1 stage, but not always, as it was also identified in wake solvers who never entered N1.

Consequently, methods that wake people up during N1 [such as a recently developed wearable electronic device called Dormio; (*37*)] have the potential to act as a creativity booster. Here, we showed that Edison’s technique seems effective at keeping subjects in the creative sweet spot by preventing deeper sleep from supplanting it. Further work, for example, using a control experiment without the bottle, would be necessary to draw definite conclusions on the effectiveness of Edison’s technique in fostering insight. Yet, because it does not require any material besides an everyday object, Edison’s technique can be applied by anyone eager to summon their creative muse, either at home or in workplaces. As our study evidenced neural signatures capturing the creativity sweet spot, closed-loop brain-computer interface methods could also be developed to help participants reach and stay within the creative sweet spot in order to boost creativity with a high level of precision. Furthermore, more research is needed to determine whether our findings are generalizable to other types of creative tasks (e.g., when the problem to solve is explicit).

We also provided empirical evidence that Edison’s technique can be used to detect subtle changes in the wake-to-sleep transition that are not captured by the traditional scoring method. Previous studies (*35*, *36*) have highlighted the difficulty of pinpointing a specific moment of sleep onset at which all measures coincide, be it electrophysiological (e.g., alpha drop, theta/delta increase, and loss of muscle tone), behavioral (e.g., cessation of responses), or subjective (e.g., hypnagogic experiences and feeling of having slept when woken up). Edison’s technique could thus help in better characterizing the fleeting moment of sleep onset by capturing each person’s specific sleep onset dynamics. Such a method could be particularly interesting in people who have a bad perception of their vigilance states (e.g., in individuals with insomnia). In addition, we showed that Edison’s technique appears optimal for collecting large amounts of hypnagogic experiences, facilitating the study of their electrophysiological signatures and specific roles in various cognitive processes (e.g., in memory).

Our study leaves open some intriguing questions for future research. For example, it is unclear why the Eureka moment occurred after around 94 trials of practicing again the task upon awakening. While this finding is fully consistent with other studies using the NRT (*1*, *12*), it contrasts with the popular view (and Edison’s story) in which a solution to a problem seemingly arises as soon as we wake up. This delayed Eureka occurred roughly 30 min after awakening and could thus correspond to the time for sleep inertia to vanish [typically less than 30 min; (*38*)]. This hypothesis is, however, not satisfactory because we did not observe any difference in the time of the Eureka moment between Wake, N1, and N2 subjects (which should increase along with an increase in sleep inertia). Furthermore, we did not find any evidence that participants used this delay as a verification stage (e.g., checking the accuracy of the hidden rule as a new strategy, which should be reflected as a slowing in solving times in the trials immediately before the Eureka moment). An alternative hypothesis is that N1 favors implicit gist abstraction by restructuring task-related memories and stores the hidden rule in a “strategy store”; then, the use of this new strategy would be determined by a second implicit process occurring when confronted again with the task during wakefulness (e.g., accumulation of evidence until a threshold determining when it is optimal to stop exploiting the original strategy and exploring whether another, more efficient strategy is available).

This explanation is, however, speculative and the suppressing effect of N2 on insight remains unexpected in this framework. One possibility is that N2 episodes were too brief to allow the full sleep-dependent memory process to unfold. By interrupting spontaneous memory reactivations of task material, we potentially destabilized them, making them more labile and susceptible to forgetting (*39*). Alternatively, it could be that N1 put participants in the creative mindset for insight, and what would matter in that case would be the last sleep stage before awakening.

Another open question is whether hypnagogic experiences play a role in inspiring insight. We did not find any evidence in favor of this hypothesis in our study. However, it is possible that some participants did not report any task-related mental experiences because they simply forgot having them or that we failed to recognize some far-fetched associations with the task because of the bizarreness of hypnagogic content (*22*).

Last, while our findings strongly support the fact that N1-related processes favor insight, it is still unclear whether some factors or individual differences facilitated the “N1 trajectory” in the first place. Further research is needed to determine the optimal conditions for hitting the creative sweet spot and whether it depends on a specific profile of individual sleep onset dynamics.

Despite its importance in our daily lives, the neural and cognitive mechanisms underlying creative problem-solving remain poorly understood. By identifying sleep onset as a key period for inspiring insight, our study provides a well-identified, short window to focus on when investigating the neural mechanisms of creative problem-solving. Overall, we hope that our work will precipitate further research into this “twilight” zone, which has been largely neglected by scientists thus far.

## MATERIALS AND METHODS

### Participants

One hundred nine participants were recruited for this study, six of whom were excluded from analysis (three for technical problems, two for sleep disorder suspicion, and one decided to prematurely stop the experiment). We thus included a total of 103 healthy participants (73 females, age 23.23 ± 3.58 years) in this study. They were screened out for exclusion criteria such as excessive daytime sleepiness, history of sleep, and neurological or psychiatric disorders. In addition, participants who fall asleep easily were selected, as measured by the Epworth scale (mean score = 9.63 ± 2.44). To further facilitate sleep onset, we asked participants to sleep about 30% less than usual during the night preceding the experiment (either by going to bed later or waking up earlier) and to avoid stimulants on the day of the experiment. Participants confirmed that they followed the sleep deprivation instruction on the day of the experiment (self-report). They were paid 10 € per hour as compensation for their participation. All subjects provided their written informed consent before the onset of the study. The study protocol was approved by the local ethics committee (Comité de Protection des Personnes, Ile-de-France III, Paris, France, approval number 2019-A00562-55).

### Behavioral task

We used the same NRT and stimuli as Craig *et al.* (*12*), but we adapted the task so that it runs on MATLAB (Psychtoolbox). Participants were presented with eight-digit strings, composed of three possible numbers (1, 4, and 9). They were told that they had to find the final solution of each string. This could be done by transforming the string into a response string, through a sequential application of two rules:

• The “same” rule: If two successive digits are the same, the response is this digit (e.g., 4-4, response: 4).

• The “different” rule: If they are different, the result is the remaining third digit (e.g., 1-4, response: 9).

To come up with the final solution, participants had to apply these rules in a stepwise manner, starting by comparing the first two digits of the string and then using their first response together with the next digit to determine the second response and so on until the end of the string. They had to press Enter to validate the final solution. Participants had no time limit, but they were told that they had to find the solution as quickly as possible. To keep them motivated, participants received feedback on whether their response was correct after each trial.

We did not mention to the subjects that a hidden regularity determined all strings. All responses' strings had the same internal structure (ABCDDCB; A, B, and C being one of the three digits 1, 4, or 9): The last three responses mirrored the preceding three responses, meaning that the second response in each trial was always the final solution. Thus, gaining insight into this regularity would markedly decrease response time because participants could abruptly shortcut their responses by pressing Enter immediately after entering the second answer. Of note, strings varied from trial to trial, thus gaining insight into the rule could not originate from the mere repetition of the same finger sequence in all trials. Correctness and solving time were recorded for each trial.

Insight was defined as:

• A steep decrease in the response time for correct trials.

• An explicit report of the hidden regularity during the posttask questionnaire.

### Experimental procedure

The protocol was subdivided into four main phases.

1) Training. Before starting the session, participants had to perform two correct strings on paper with the experimenter to ensure that they correctly understood the task. They then started the computerized task with the training phase composed of 10 trials (that did contain the hidden rule).

2) Pre phase. Following training, they had to complete two blocks of 30 trials each.

3) Break. All participants had a 20-min break in a dark room without sensory stimulation. They were installed in a semi-reclined position in a chair, with their eyes closed and legs on a footrest. They had to hold an object in their right hand, with their hand carefully placed outside the armrest (see Fig. 1B). Participants were simply told to relax or sleep if desired. If the object fell (the sound of which awakened them), they were instructed to describe out loud what was going through their mind before it fell. We told them that their mental content could be of any kind: thoughts, images, reveries, and dreams. Once done, they were told to pick up the object and repeat this procedure until the end of the break. Participants were constantly monitored with an infrared camera to ensure that they followed the instructions appropriately.

At the end of the rest period, we asked participants to describe their mental content during the break and state whether they thought about the task, whether they were awake or drowsy, and whether holding the object was tiring and/or preventing them from falling asleep. This design allowed us to naturally subdivide our subjects into three main groups:

• Wake group (subjects who did not sleep): *N* = 49

• N1 group (subjects who entered N1, without any signs of other sleep stages): *N* = 24

• N2 group (subjects who attained the second stage N2): *N* = 14

4) Post phase. Then, approximately 10 min after awakening (a time interval that included a questionnaire on hypnagogia, the instructions, and a PVT), they performed the Post session, which consisted of nine blocks of 30 trials each (always containing the regularity) and lasted 64 min on average.

To control for any groups' differences in the vigilance state, participants also performed a 3-min PVT (*40*) at the beginning of the Pre and Post phases. Here, participants had to respond to visual stimuli as quickly and accurately as possible (intertrial interval varying between 1 and 4 s including a 1-s-feedback screen), and performance was measured as the average reaction time to the appearance of the visual cue. As additional controls, we also assessed their subjective levels of sleepiness, boredom, concentration, and motivation on a five-point scale (as in Yordanova *et al.*) (*41*) before and after the Pre and Post sessions. At the end of the experiment, we verified their knowledge of the rule and asked whether they were used to solve enigmas.

### Object

We tested many objects (spoon, small steel spheres, stress balls, etc.) before finding the right one. We determined that the ideal object needed to meet the following criteria: makes a noise when falling, light weight to avoid cramped arms, slippery to facilitate the fall, and large enough so that the fist cannot close on it (and prevents the object from falling). The object that we chose for this experiment was a 14.5-cm-tall, 5.5-cm-diameter, light (55 g) drinking cup/bottle (see Fig. 1B), which succeeded in capturing sleep onset in a pilot study. This object is cheap (around 3€) and anyone who wishes to buy one can ask us for the fabric.

### EEG recordings

Subjects were continuously monitored with video polysomnography during the experiment. The montage included three EEG channels (FP1, C3, and O1), EOG with electrodes placed on the outer canthi of the eyes, chin EMG, a microphone, and infrared video recordings (Brainet, Medatec Ltd., France). Impedances of electrodes were generally below 5 kilohms. EEG signals were referenced to A2 (right mastoid) and sampled at 250 Hz.

### Sleep scoring

EEG signals were band pass–filtered between 0.1 and 40 Hz and EOG signals were band pass–filtered between 0.3 and 15 Hz (two-pass Butterworth filter, fifth order). EMG signal was obtained with a local derivation and band pass–filtered between 10 and 100 Hz (two-pass Butterworth filter, fifth order). Vigilance state of participants during the break was scored offline by two experienced scorers (C.L. and D.O.), blind to experimental conditions. The interrater agreement was very good (Cohen’s κ coefficient = 0.83), and remaining disagreements were examined by a third expert scorer (S. Leu).

Two scoring methods were applied:

• The standard sleep scoring guidelines from the American Academy of Sleep Medicine (AASM) (*34*). Any subject with at least one epoch of N1 was assigned to the N1 group, unless signs of N2 (spindles, K complexes) were present (in that case, the subject was put in the N2 group). By definition, no participant from the Wake group had more than 15 s of continuous sleep (standard criterion to score an epoch in sleep), but they could have had shorter episodes of sleep.

• A microsleep scoring method, similar to the one recently developed by Hertig-Godeschalk *et al.* (*42*). EEG was scored continuously to identify MSEs, which were defined here as any short window of at least 3 s of theta activity with alpha loss. This method was used to detect MSEs that are not captured by the standard sleep scoring method, providing an in-depth analysis of the wake-to-sleep transition zone, which is the focus of the present paper.

### The Eureka moment

To detect sudden changes in solving time (taken here as a marker of insight), we used an algorithm provided by MATLAB named “findchangepts”, which detects abrupt changes in signals. For a vector x with N elements, the “findchangepts” function partitions x into two regions, x(1:changepoint-1) and x(changepoint:N), which minimizes the sum of the residual (squared) error of each region from its local mean. The same algorithm was used for both solvers and nonsolvers. By definition, the algorithm always finds a point in which solving time decreases the most even if the “drop” is small or corresponds to a random, not sustainable deviation in reaction time in one trial, which was typically the case in nonsolvers.

### Hypnagogic experiences

We compared the proportion of hypnagogic experiences collected right after the bottle drop and at the end of the rest period (for participants who did not drop the bottle). Here, a mental report was considered a hypnagogic experience only if it was a “fleeting, involuntary, spontaneous, perceptual, and bizarre mental content” (some examples are reported in the Supplementary Materials, see text and video). Of note, if we had used a broader definition (i.e., any reported mentation), the percentage of reported hypnagogic experiences at drop would have been 100%, rather than 63.64% with our more conservative definition (see Results). To account for the time factor in hypnagogic recall, we also compared the amount of reported hypnagogic experiences at the bottle drop with the one obtained in an additional control experiment, in which subjects took a 30-min break in a dark bedroom; they were regularly awakened by a sound and asked to describe their mental content. We then calculated the percentage of reported hypnagogia when the awakening sound occurred after MSEs to be in comparable conditions to the current study.

### EEG spectral analyses

We computed the power spectrum of EEG electrode O1 in two different ways. First, we extracted the power spectrum over contiguous epochs of 30 s (corresponding to the epochs used for sleep scoring). We used Welch's method to compute the power spectra density between 1 and 30 Hz using windows of 6 s with 50% overlap and a frequency resolution of 0.2 Hz. The power over each 6-s window was averaged for each 30-s epoch. Epochs that exceeded an absolute amplitude of 150 μV were excluded from this analysis. We then applied the FOOOF toolbox to compute a smoothed version of the power spectrum (*43*). The corresponding power spectra were finally averaged across the entire break. Second, we computed the time-resolved power spectrum around bottle drops and random time drops (−50 to 10 s around these times). Windows that exceeded an absolute amplitude of 750 μV before a drop were excluded from this analysis (7 of 46 recordings). We used a multitaper time-frequency decomposition with a single Hanning taper over 6-s-long windows. Power was extracted between 1 and 30 Hz with a 0.2-Hz resolution. This operation was performed for each bottle drop in each recording (if any), and the corresponding power was averaged across drops where a recording contained more than one. For comparison, we also generated random time drops using a uniform distribution between the beginning and end of the break. A total of 100 random times were drawn for each recording with at least one drop, and power was averaged across these draws.

### Statistical analyses

Interjudges’ agreement was evaluated with the Cohen’s Κ test. Fisher's and chi-square tests were used in the analysis of contingency tables. Kruskal-Wallis tests were used for ordinal variables. Nonparametric statistics were applied (Mann-Whitney *U* test for independent samples, Wilcoxon signed-rank test for paired samples, and Kruskal-Wallis test for more than two groups) when variables could not be approximated to the normal distribution (Shapiro-Wilk test). All tests were two-tailed, and a probability level of less than 0.05 was considered significant. All computations were performed using MATLAB 2018b. For the analysis of the power spectra (Fig. 3A), we applied two-way analysis of variance (ANOVA) at each frequency bin with two between-subject predictors: sleep (whether participants fell asleep during the break or not) and insight (whether participants found the solution after the break or not). The power (predicted variable) was either *z*-scored (across recordings) at each frequency bin to investigate linear effects of the predictors or *z*-scored and squared to examine quadratic effects. To correct for multiple comparisons, we used a cluster permutation approach (*43*) with a cluster alpha set at 0.1 (used to define the candidate clusters), a Monte Carlo alpha set at 0.05 (used to determine the significance of the clusters), and 1,000 permutations. A minimal size of five consecutive frequency bins was applied for significant clusters. This stringent approach is designed to control for type I errors (false positives) and resulted in two clusters with narrow frequency bands. While this method allows concluding on the existence of statistically significant differences, it does not permit the absolute demarcation of these effects (*44*). Thus, our findings do not affirm that the effects are restricted to the narrow bands identified by the cluster permutation. In addition, only the effect of insight (linear and quadratic) is shown in Fig. 3A. Power was then averaged for each of the clusters obtained, and a two-way ANOVA, like the ones described above, was applied to the *z*-scored and squared *z*-scored power (Fig. 3, B and D).

We also fitted logistic regression models to attempt to predict insight with a set of variables of interest (sleep group, *z*-scored delta power, *z*-scored squared delta power, *z*-scored alpha power, and *z*-scored squared alpha power) and covariates (bottle drop, Epworth score, mean post PVT reaction times, and percentage of microsleep). We fitted models with increasing levels of complexity as follows:

Model 0: *Insight* ~1

Model 1: *Insight* ~1 + *Sleep_Group*

Model 2: *Insight* ~1 + *Sleep_Group* + *Delta_Power*

Model 3: *Insight* ~1 + *Sleep_Group* + *Delta_Power* + *Alpha_Power*^2^

Model 4: *Insight* ~1 + *Sleep_Group* + *Delta_Power* + *Alpha_Power*^2^ + *Alpha_Power*

Model 5: *Insight* ~1 + *Sleep_Group* + *Delta_Power* + *Alpha_Power*^2^ + *Alpha_Power* + *Delta_Power*^2^

Model 6: *Insight* ~1 + *Sleep_Group* + *Delta_Power* + *Alpha_Power*^2^ + *Alpha_Power* + *Delta_Power*^2^ + *PVT_RT* + *Bottle_Drop* + *Epworth_Score* + *Micro_Sleep*

Generalized linear models were fitted with the glm function from the “stats” package in R. We examined the AIC to determine which model fitted the data best (AIC for models 0 to 6: 109.3, 103.2, 100.5, 94.4, 95.9, 97.2, and 102.1). Accordingly, model 3 was the model that provided the best fit. The only model within two AIC units of the best model was model 4. We report the effects associated with model 3 in Results and table S2.

## Supplementary Materials

### This PDF file includes:

Supplementary Text Figs. S1 and S2 Tables S1 and S2 Legend for movie S1 References

## Notes

View/request a protocol for this paper from *Bio-protocol*.
