# Supplementary material for: Sleep onset is a creative sweet spot
Source: Sci Adv. 2021 Dec 8;7(50):eabj5866. doi: 10.1126/sciadv.abj5866 (PMC8654287; doi:10.1126/sciadv.abj5866)
Supplement: Supplementary file 1 — Supplementary Text Figs. S1 and S2 Tables S1 and S2 Legend for movie S1 References [file sciadv.abj5866_sm.pdf]

Science Advances
AAAS

Supplementary Materials for

Sleep onset is a creative sweet spot

Célia Lacaux, Thomas Andrillon, Céleste Bastoul, Yannis Idir, Alexandrine Fonteix-Galet, Isabelle Arnulf, Delphine Oudiette*

*Corresponding author. Email: delphine.oudiette@icm-institute.org

Published 8 December 2021, *Sci. Adv.* **7**, eabj5866 (2021)
DOI: 10.1126/sciadv.abj5866

The PDF file includes:

Supplementary Text
Figs. S1 and S2
Tables S1 and S2
Legend for movie S1
References

Other Supplementary Material for this manuscript includes the following:

Movie S1

Supplementary Text

Examples of hypnagogic reports

- “I saw geometric shapes, like the old screensavers on computers where there was a square that became a triangle, then a round. Something like that, blurry, with colours.”
- “I saw lots of numbers in black with little triangles. At first, they were far away, and then they came closer. Then, it was calmer, the sea, with little boats in the sand. And then I saw the strings of my guitar and I played piano with the keyboard.”
- “I was in wheat fields.”
- “At one point, I saw a horse in the hospital. There was also a man who was doing the same experiment as me, who was much older and who had a kind of plastic helmet on his face.”
- “I was hearing a sound that said 1,4, 9.”
- “I had the feeling of being at the water's edge, no wind, there were airy sounds, as in a temperate forest in summer.”
- “I was inside the Colosseum in Rome when it was still in its prime. I also saw concentric shapes that were shrinking and certain figures that I called functions that I was trying to identify. One that really struck me was what I called the oscillator that turned one figure into another and then back to the original one.”

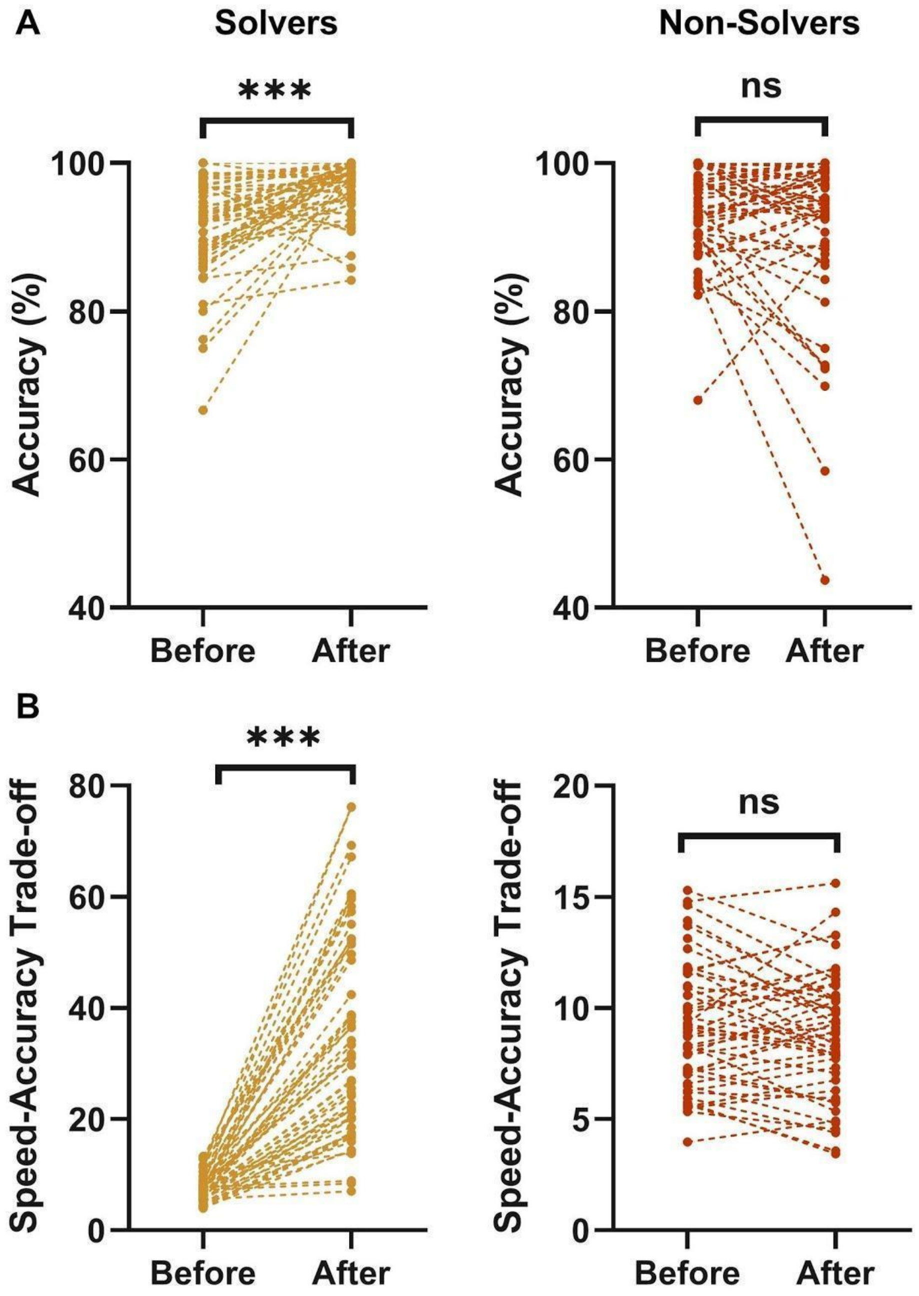

Fig. S1. Performances before and after Eureka. *Accuracy* ***(A)*** *and speed-accuracy trade-off* ***(B)*** *prior and after "Eureka" for all solvers (left panel, N = 53) and non-solvers (right panel, N = 50). ***, p <0.001; n.s. for non-significant differences (Wilcoxon signed-rank for comparisons between two paired samples).*

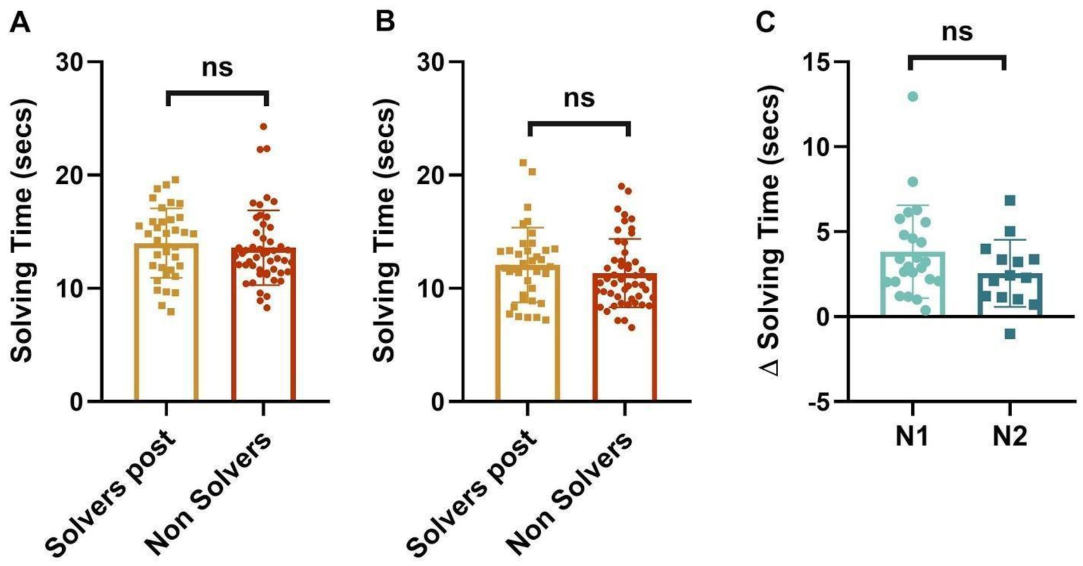

***Fig. S2. No evidence for an implicit knowledge before Eureka** Mean Solving Time on the last block of the Pre phase **(A),** and on the Post phase until the Eureka moment **(B)** for both solvers post and non-solvers (here Eureka was approximated as the mean Eureka moment of solvers post, namely trial 94). **(C)** Difference in solving time (last block of pre minus first block of post) for N1 and N2 groups. n.s. for non-significant differences between groups (Wilcoxon).*

	Wake	N1	N2	P
**Demographic data**				
Age	23.22 ± 3.57	23.17 ± 3.56	24.79 ± 4.49	0.43
Laterality (right-handed), %	81.63	79.17	78.57	0.95
Gender, women, %	77.55	58.33	85.71	0.12
Educational level	6.65 ± 1.57	6.63 ± 1.82	6.79 ± 1.22	0.41
Epworth Score	9.47 ± 2.65	9.21 ± 1.69	9.14 ± 2.21	0.56
Field of expertise (1-4)	2.02 ± 1.22	2.04 ± 1.04	2.08 ± 1.0	0.86
**Cofactors**				
Accuracy *Pre*, %	94.51	92.78	93.69	0.76
Solving Time *Pre*, secs	13.93	15.24	15.21	0.24
PVT RT *Pre*, msecs	303.3 ± 58.9	294.3 ± 35.2	312.8 ± 36.1	0.23
PVT RT *Post*, msecs	281.1 ± 26.4	281.7 ± 30.4	304.4 ± 38.5	**0.03+**
Motivation (1-5)	3.43 ± 1.08	3.21 ± 1.24	3.08 ± 1.19	0.49
Sleepiness (1-5)	2.50 ± 1.07	2.90 ± 0.85	2.58 ± 1.00	0.40
Concentration (1-5)	3.29 ± 0.76	3.04 ± 1.05	3.31 ± 0.85	0.57
Boredom (1-5)	1.84 ± 0.92	2.02 ± 1.04	2.58 ± 0.81	**0.03+**
Enigma expertise (1-3)	1.37 ± 0.67	1.54 ± 0.72	1.29 ± 0.61	0.36
**Sleep data**				
Wake duration, min	21.60 ± 1.19	20.15 ± 1.76	15.43 ± 4.28	**<0.001$**
N1 duration, min	0 ± 0	1.35 ± 1.20	1.79 ± 2.11	**<0.001***
N2 duration, min	0 ± 0	0 ± 0	4.18 ± 2.94	**<0.001#**
Sleep latency, min	NA	14.46 ± 4.45	11.18 ± 5.04	**0.04**
**MSE data**				
MSE duration, min	0.16 ± 0.25	1. 90 ± 1.13	2.30 ± 1.89	**<0.001***
Wake-to-MSE transitions, N	2.16 ± 3.50	14.29 ± 9.82	15.57 ± 10.96	**<0.001***
**Bottle data**				
% of subjects with drop	34.69	79.17	78.57	**<0.001***
MSE duration before fall, secs	3.0 ± 6.38	64.77 ± 43.51	75.11 ± 61.89	**<0.001***
MSE bouts before fall, N	0.83 ± 1.79	8.35 ± 6.24	9.16 ± 6.46	**<0.001***
Last MSE before fall, secs	1.39 ± 2.17	10.31 ± 7.28	8.21 ± 6.8	**<0.001***

Table S1. Demographic and sleep characteristics of each group. *Measures are presented as the mean ± the standard deviation or in percentages.* $N_{Wake} = 49$, $N_{N1} = 24$, $N_{N2} = 14$. *Educational*

level is scored according to the International Standard Classification of Education (*45*). *Fields of expertise are categorized as follows: 1 for Sciences, 2 for Humanities, 3 for Arts, and 4 for Others. Enigma expertise is self-reported with 1 for No, 2 for A little, and 3 for Yes. Subjective ratings of motivation, sleepiness, concentration, and boredom were collected at the start of the Post phase. MSE stands for Micro-Sleep Episodes, and MSE duration refers to MSE cumulated time. p-values are displayed (ANOVA or Kruskal-Wallis Tests when appropriate: ordinal data or violation of normality assessed with the Shapiro-Wilk test) and significant differences between groups are highlighted in bold. When appropriate, post-hoc comparisons (Tukey-Kramer) have been computed and we report significant difference between Wake and N2 (+), Wake and N1/N2 (*), N2 and Wake/N1 (#), and between all groups ($).*

**Coefficient**	**Estimate**	**Standard-Error**	**Chi-squared**	**P-Value**
**Sleep Group**	-0.64	0.27	6.24	**0.01 (*)**
**Delta_Power**	-0.71	0.31	5.99	**0.01 (*)**
**Alpha_Power^2**	-0.72	0.30	8.11	**0.004 (**)**

Table S2. Predicting insight with pre-nap, nap and post-nap features. Estimates, Standard Errors, Chi-Squared and P-values for Model 3 (Insight ~ 1 + Sleep_Group + Delta_Power + Alpha_Power^2), the best fitting model obtained in our analyses. Significant effects are highlighted in bold fonts.

Supplementary Video. **Examples of hypnagogic experiences reported by participants.** Participants reported their mental content either directly after dropping the bottle and at the end of the break.

REFERENCES AND NOTES

1. U. Wagner, S. Gais, H. Haider, R. Verleger, J. Born, Sleep inspires insight. *Nature* **427**, 352–355 (2004).

2. L. Batterink, D. Oudiette, P. Reber, K. Paller, Sleep facilitates learning a new linguistic rule. *Neuropsychologia* **65**, 169–179 (2014).

3. S. J. Durrant, C. Taylor, S. Cairney, P. A. Lewis, Sleep-dependent consolidation of statistical learning. *Neuropsychologia* **49**, 1322–1331 (2011).

4. V. L. Perdomo, W. F. Hofman, L. M. Talamini, Sleep fosters insight into real-life problems. *Arch. Ital. Biol.* **156**, 87–98 (2018).

5. K. E. G. Sanders, S. Osburn, K. A. Paller, M. Beeman, Targeted memory reactivation during sleep improves next-day problem solving. *Psychol. Sci.* **30**, 1616–1624 (2019).

6. D. J. Cai, S. A. Mednick, E. M. Harrison, J. C. Kanady, S. C. Mednick, REM, not incubation, improves creativity by priming associative networks. *Proc. Natl. Acad. Sci. U.S.A.* **106**, 10130–10134 (2009).

7. R. Stickgold, L. Scott, C. Rittenhouse, J. A. Hobson, Sleep-induced changes in associative memory. *J. Cogn. Neurosci.* **11**, 182–193 (1999).

8. M. P. Walker, C. Liston, J. A. Hobson, R. Stickgold, Cognitive flexibility across the sleep-wake cycle: REM-sleep enhancement of anagram problem solving. *Brain Res. Cogn. Brain Res.* **14**, 317–324 (2002).

9. N. Landmann, M. Kuhn, H. Piosczyk, B. Feige, C. Baglioni, K. Spiegelhalder, L. Frase, D. Riemann, A. Sterr, C. Nissen, The reorganisation of memory during sleep. *Sleep Med. Rev.* **18**, 531–541 (2014).

10. P. A. Lewis, G. Knoblich, G. Poe, How memory replay in sleep boosts creative problem-solving. *Trends Cogn. Sci.* **22**, 491–503 (2018).

11. J. G. Klinzing, N. Niethard, J. Born, Mechanisms of systems memory consolidation during sleep. *Nat. Neurosci.* **22**, 1598–1610 (2019).

12. M. Craig, G. Ottaway, M. Dewar, Rest on it: Awake quiescence facilitates insight. *Cortex* **109**, 205–214 (2018).

13. T. Horikawa, M. Tamaki, Y. Miyawaki, Y. Kamitani, Neural decoding of visual imagery during sleep. *Science* **340**, 639–642 (2013).

14. E. Tagliazucchi, H. Laufs, Decoding wakefulness levels from typical fMRI resting-state data reveals reliable drifts between wakefulness and sleep. *Neuron* **82**, 695–708 (2014).

15. R. E. Beaty, M. Benedek, P. J. Silvia, D. L. Schacter, Creative cognition and brain network dynamics. *Trends Cogn. Sci.* **20**, 87–95 (2016).

16. H. Laufs, J. L. Holt, R. Elfont, M. Krams, J. S. Paul, K. Krakow, A. Kleinschmidt, Where the BOLD signal goes when alpha EEG leaves. *Neuroimage* **31**, 1408–1418 (2006).

17. D. Picchioni, M. Fukunaga, W. S. Carr, A. R. Braun, T. J. Balkin, J. H. Duyn, S. G. Horovitz, fMRI differences between early and late stage-1 sleep. *Neurosci. Lett.* **441**, 81–85 (2008).

18. D. Foulkes, G. Vogel, Mental activity at sleep onset. *J. Abnorm. Psychol.* **70**, 231–243 (1965).

19. G. W. Vogel, B. Barrowclough, D. D. Giesler, Limited discriminability of REM and sleep onset reports and its psychiatric implications. *Arch. Gen. Psychiatry* **26**, 449–455 (1972).

20. R. Stickgold, A. Malia, D. Maguire, D. Roddenberry, M. O'Connor, Replaying the game: Hypnagogic images in normals and amnesics. *Science* **290**, 350–353 (2000).

21. E. J. Wamsley, M. Tucker, J. D. Payne, J. A. Benavides, R. Stickgold, Dreaming of a learning task is associated with enhanced sleep-dependent memory consolidation. *Curr. Biol.* **20**, 850–855 (2010).

22. P. Stenstrom, K. Fox, E. Solomonova, T. Nielsen, Mentation during sleep onset theta bursts in a trained participant: A role for NREM stage 1 sleep in memory processing? *Int. J. Dream Res.* **5**, 37–46 (2012).

23. T. Andrillon, J. Windt, T. Silk, S. P. A. Drummond, M. A. Bellgrove, N. Tsuchiya, Does the mind wander when the brain takes a break? Local sleep in wakefulness, attentional lapses and mind-wandering. *Front. Neurosci.* **13**, 949 (2019).

24. K. Christoff, Z. C. Irving, K. C. R. Fox, R. N. Spreng, J. R. Andrews-Hanna, Mind-wandering as spontaneous thought: A dynamic framework. *Nat. Rev. Neurosci.* **17**, 718–731 (2016).

25. B. Baird, J. Smallwood, M. D. Mrazek, J. W. Y. Kam, M. S. Franklin, J. W. Schooler, Inspired by distraction: Mind wandering facilitates creative incubation. *Psychol. Sci.* **23**, 1117–1122 (2012).

26. C. Lacaux, C. Izabelle, G. Santantonio, L. De Villèle, J. Frain, T. Lubart, F. Pizza, G. Plazzi, I. Arnulf, D. Oudiette, Increased creative thinking in narcolepsy. *Brain* **142**, 1988–1999 (2019).

27. A. D'Anselmo, S. Agnoli, M. Filardi, F. Pizza, S. Mastria, G. E. Corazza, G. Plazzi, Creativity in narcolepsy type 1: The role of dissociated REM sleep manifestations. *Nat. Sci. Sleep* **12**, 1191–1200 (2020).

28. M. Magnin, M. Rey, H. Bastuji, P. Guillemant, F. Mauguière, L. Garcia-Larrea, Thalamic deactivation at sleep onset precedes that of the cerebral cortex in humans. *Proc. Natl. Acad. Sci. U.S.A.* **107**, 3829–3833 (2010).

29. R. D. Ogilvie, R. T. Wilkinson, The detection of sleep onset: Behavioral and physiological convergence. *Psychophysiology* **21**, 510–520 (1984).

30. M. H. Bonnet, S. E. Moore, The threshold of sleep: Perception of sleep as a function of time asleep and auditory threshold. *Sleep* **5**, 267–276 (1982).

31. T. Hori, M. Hayashi, T. Morikawa, in *Sleep Onset: Normal and Abnormal Processes* (American Psychological Association, 1994), pp. 237–253.

32. A. J. H. Horowitz, "Incubating Dreams, Awakening Creativity," thesis, Massachusetts Institute of Technology (2019).

33. L. L. Thurstone, T. G. Thurstone, Factorial studies of intelligence. *Psychom. Monogr.* **2**, 94 (1941).

34. R. B. Berry, R. Brooks, C. E. Gamaldo, S. M. Harding, C. Marcus, B. V. Vaughn, The AASM manual for the scoring of sleep and associated events. *Rules Terminol. Tech. Specif. Darien Ill. Am. Acad. Sleep Med.* **176**, 2012 (2012).

35. R. D. Ogilvie, The process of falling asleep. *Sleep Med. Rev.* **5**, 247–270 (2001).

36. M. J. Prerau, K. E. Hartnack, G. Obregon-Henao, A. Sampson, M. Merlino, K. Gannon, M. T. Bianchi, J. M. Ellenbogen, P. L. Purdon, Tracking the sleep onset process: An empirical model of behavioral and physiological dynamics. *PLoS Comput. Biol.* **10**, e1003866 (2014).

37. A. Horowitz, T. J. Cunningham, P. Maes, R. Stickgold, Dormio: A targeted dream incubation device. *Conscious. Cogn.* **83**, 102938 (2020).

38. L. M. Trotti, Waking up is the hardest thing I do all day: Sleep inertia and sleep drunkenness. *Sleep Med. Rev.* **35**, 76–84 (2017).

39. K. Nader, O. Hardt, A single standard for memory: The case for reconsolidation. *Nat. Rev. Neurosci.* **10**, 224–234 (2009).

40. D. A. Grant, K. A. Honn, M. E. Layton, S. M. Riedy, H. P. A. Van Dongen, 3-minute smartphone-based and tablet-based psychomotor vigilance tests for the assessment of reduced alertness due to sleep deprivation. *Behav. Res. Methods* **49**, 1020–1029 (2017).

41. J. Yordanova, V. Kolev, U. Wagner, R. Verleger, Differential associations of early- and late-night sleep with functional brain states promoting insight to abstract task regularity. *PLOS ONE* **5**, e9442 (2010).

42. A. Hertig-Godeschalk, J. Skorucak, A. Malafeev, P. Achermann, J. Mathis, D. R. Schreier, Microsleep episodes in the borderland between wakefulness and sleep. *Sleep* **43**, zsz163 (2020).

43. T. Donoghue, J. Dominguez, B. Voytek, Electrophysiological frequency band ratio measures conflate periodic and aperiodic neural activity. *eNeuro* **7**, ENEURO.0192-20.2020 (2020).

44. E. Maris, R. Oostenveld, Nonparametric statistical testing of EEG- and MEG-data. *J. Neurosci. Methods* **164**, 177–190 (2007).

45. S. L. Schneider, in *Class and Stratification Analysis* (Emerald Group Publishing Limited, 2013), vol. 30 of *Comparative Social Research*, pp. 365–379.
